# Supplementary material for: The Influence of Synthesis Method on Characteristics of Buffer and Organic Solutions of Thermo- and pH-Responsive Poly(N-[3-(diethylamino)propyl]methacrylamide)s
Source: Polymers (Basel). 2022 Jan 11;14(2):282. doi: 10.3390/polym14020282 (PMC8777966; doi:10.3390/polym14020282)
Supplement: Supplementary file 1 [file polymers-14-00282-s001.zip › polymers-1496649-supplementary.pdf]

Supplementary materials

The Influence of Synthesis Method on Characteristics of Buffer and Organic Solutions of Thermo- and pH-Responsive Poly(N-[3-(diethylamino) propyl]methacrylamide)s

Maria Simonova [1,*], Denis Kamorin [2,3], Anton Sadikov [2], Alexander Filippov [1], and Oleg Kazantsev [2]

[1] Institute of Macromolecular Compounds of the Russian Academy of Sciences, Bolshoy Prospekt 31, 199004 Saint Petersburg, Russia; afil@imc.macro.ru

[2] Laboratory of Acrylic Monomers and Polymers, Department of Chemical Technology, Dzerzhinsk, Polytechnic Institute, Nizhny Novgorod State Technical University n.a. R.E. Alekseev, 24 Minin Street, 603950 Nizhny Novgorod, Russia; d.kamorin@mail.ru (D.K.); mr.sadikovanton@mail.ru (A.S.); kazantsev@dpingtu.ru (O.K.)

[3] Chromatography Laboratory, Department of Production Control and Chromatography Methods, Lobachevsky State University of Nizhni Novgorod, Dzerzhinsk Branch, 23 Prospekt Gagarina, 603950 Nizhny Novgorod, Russia

* Correspondence: mariasimonova1983@mail.ru; Tel.: +7-81-2328-4102

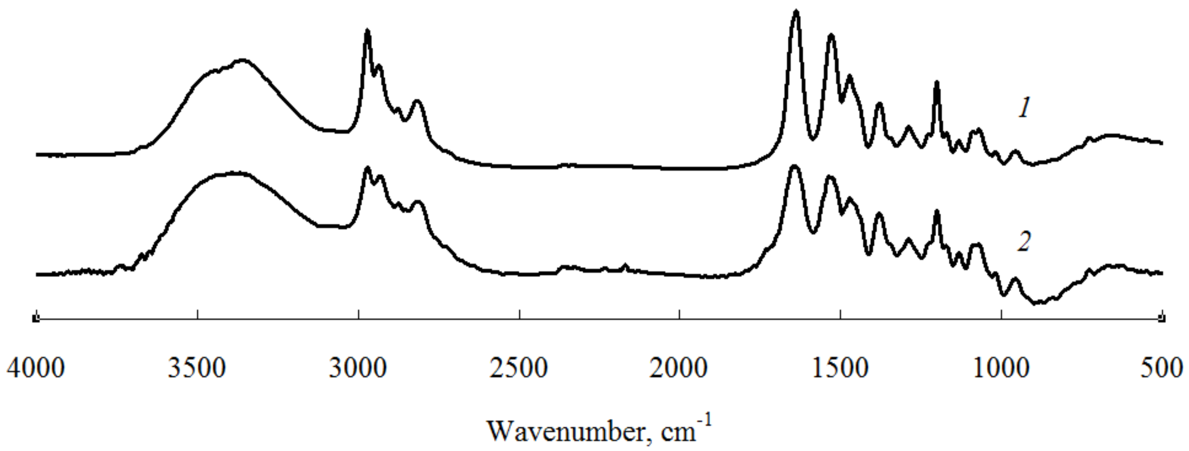

Figure S1. IR spectra of the PDEAPMA-C (*1*) and PDEAPMA-R (*2*).

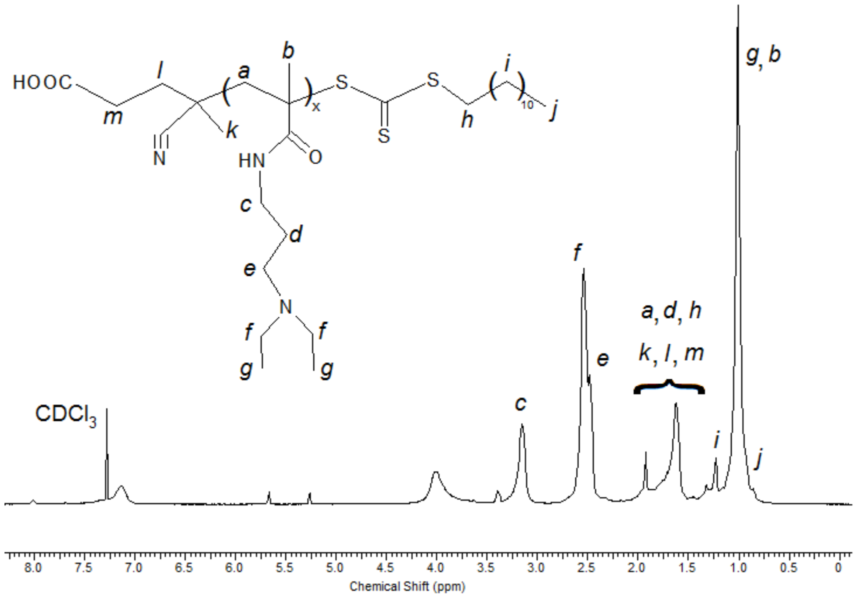

Figure S2. ^{1}H NMR spectrum of the PDEAPMA-R.

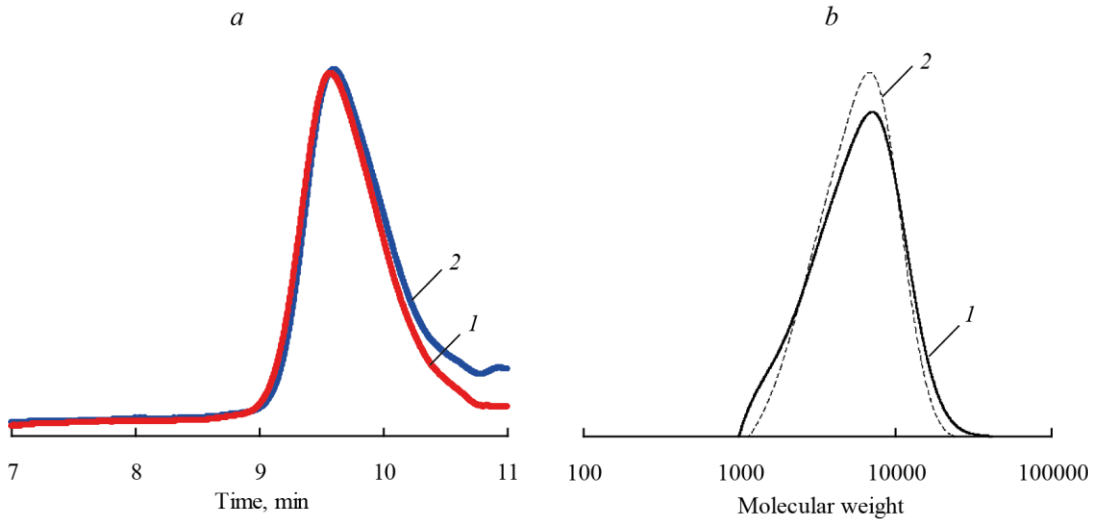

Figure S3. Figure S3: SEC traces (*a*) and molecular weight distribution (*b*) for the PDEAPMA-C (1) and PDEAPMA-R (2).
